# Emerging organisational models of primary healthcare and unmet needs for care: insights from a population-based survey in Quebec province

**DOI:** 10.1186/1471-2296-13-66

**Published:** 2012-07-02

**Authors:** Jean-Frédéric Levesque, Raynald Pineault, Marjolaine Hamel, Danièle Roberge, Costas Kapetanakis, Brigitte Simard, Alexandre Prud’homme

**Affiliations:** 1 Institut national de santé publique du Québec, 190 boulevard Crémazie Est, Montréal, Québec, Canada; 2 Direction de santé publique de Montréal, 190 boulevard Crémazie Est, Montréal, Québec, Canada; 3 Centre de recherche du Centre hospitalier, de l'Université de Montréal, Montréal, Québec, Canada; 4 Centre de recherche, de l'hôpital Charles-Lemoyne, Montréal, Québec, Canada

**Keywords:** Primary care, Unmet needs for care, Primary healthcare organization, Vulnerability

## Abstract

**Background:**

Reform of primary healthcare (PHC) organisations is underway in Canada. The capacity of various types of PHC organizations to respond to populations’ needs remains to be assessed. The main objective of this study was to evaluate the association of PHC affiliation with unmet needs for care.

**Methods:**

Population-based survey of 9205 randomly selected adults in two regions of Quebec, Canada. Outcomes Self-reported unmet needs for care and identification of the usual source of PHC.

**Results:**

Among eligible adults, 18 % reported unmet needs for care in the last six months. Reasons reported for unmet needs were: waiting times (59 % of cases); unavailability of usual doctor (42 %); impossibility to obtain an appointment (36 %); doctors not accepting new patients (31 %). Regression models showed that unmet needs were decreasing with age and was lower among males, the least educated, and unemployed or retired. Controlling for other factors, unmet needs were higher among the poor and those with worse health status. Having a family doctor was associated with fewer unmet needs. People reporting a usual source of care in the last two-years were more likely to report unmet need for care. There were no differences in unmet needs for care across types of PHC organisations when controlling for affiliation with a family physician.

**Conclusion:**

Reform models of primary healthcare consistent with the medical home concept did not differ from other types of organisations in our study. Further research looking at primary healthcare reform models at other levels of implementation should be done.

## Background

From the perspective of users, unmet needs for care have been defined as perceived needs for receiving healthcare services that are not obtained [1]. Unmet needs are associated with factors related to both the availability of care and individual characteristics [2]. In the Canadian context, the reporting of unmet needs among population surveys shows a steady increase. From levels as low as 4 % in 1994–1995 and 6 % in 1998–1999, the most recent survey suggests the prevalence of unmet needs for care to be between 8 and 13 % [3-5]. Other studies have estimated the prevalence of unmet needs for care to range from 8 % in Australia, 10 % in Canada and in the United Kingdom, to up to 12 % and 14 % respectively in New Zealand and the United States over a period of 12 months [6,7]. When delays are included in the definition of unmet needs for care, up to 25 % of citizens of the United States report occurrence in a one year period [8,9].

These studies have highlighted the association of certain individual and household characteristics with the occurrence of unmet needs for care (see Additional file 1: Appendix for details). In general, females, younger individuals, those with higher education, and recent immigrants tend to report higher levels of unmet needs for care [1,9-14]. With regards to economic status, studies have tended to show different results depending on the specific context where they were done. Studies from countries with universal tax-based or social insurance health system coverage tend to show little relationship between unmet needs for care and economic status [10]. In contrast, studies conducted in the United States show a gradient relationship between unmet needs for care and lower economic status [8,12].

The impact of perceived health and chronic illness on the reporting of unmet healthcare needs has also been documented. People perceiving themselves as being in poorer health status and those reporting a chronic illness tend to report more unmet needs for care [8,9,11,14]. Finally, studies have suggested that having a regular source of care or a family doctor is associated with reduced occurrence of unmet needs for care [10,12]. These different characteristics can affect unmet needs through their association with perception of need for care, knowledge about where to obtain services, as well as other barriers related to access to care such as an ability to reach required services and to pay for care.

The increase in unmet needs for care parallels identified problems with regards to access to primary healthcare, as highlighted by various federal and provincial commissions in Canada [15-18]. These commissions have emphasized the crucial role of primary healthcare on the overall performance of healthcare systems and have advocated the development of new delivery models. In the province of Quebec, the current reform model consists of the implementation of Family medicine groups and the integration of services locally through the establishment of local health and social service networks [19]. Family medicine groups consist of six to twelve family physicians working together with one or two nurses, administrative support technicians, extended opening hours during evenings and weekends, and telephone access to a specific roster of patients.

This new model of primary healthcare is consistent with recent proposals to implement the concept of the patient-centred medical home (PCMH) into primary healthcare. The PCMH is often described as the vision toward which primary care organizations should ideally be converging [20,21]. This concept is now internationally considered to be a model that improves primary care and patients’ access to services. The PCMH is defined as a care setting where: 1) patients have a personal family physician who provides and directs their medical care; 2) care is for the whole person’s needs; 3) care is coordinated, continuous, and comprehensive with patients having access to an inter-professional team; 4) there is enhanced access for appointments; 5) the practice includes well-supported information technology, including an electronic medical record; 6) remuneration supports the model of care; and 7) quality improvement and patient safety are key objectives. This approach is based on the existence of an ongoing relationship between the patient and the family doctor [22].

However, the capacity of these new models of care as well as that of current delivery models to meet populations need for care or to reduce barriers to access to healthcare have not been studied. In addition, little information is available on the reasons for experiencing unmet healthcare needs and their consequences on people. This study aims to fill this gap by assessing the occurrence, correlates, reasons, and consequences of unmet healthcare needs in a population-based sample. More specifically, this study’s objective is to assess the impact of primary care affiliation on the reporting of unmet needs for care.

## Methods

A representative sample of the household-dwelling population of Montreal and Montérégie regions of Québec province in Canada was drawn using the Random Digit Dialling methods between February and June 2005. One adult was randomly selected in each household. Respondents unable to respond in French or English were excluded. A total of 9205 adults accepted to participate^a^ (response rate of 64 %).^b^ In this study, as in previous studies [1,6,8,9,12,13], we have defined unmet needs for care as “Perceived need for receiving health care services that are not obtained.” A 6 month recall period was selected to reduce recall bias. An index of morbidity was used by assessing the absence of health problems, the occurrence of isolated risk factors (high blood pressure, high blood cholesterol, diabetes), the prevalence of moderate morbidities (various conditions), and of severe morbidities (cardiovascular diseases, heart failure, chronic obstructive pulmonary diseases). A standardised questionnaire, derived from a review of the questions found in the scientific literature on unmet needs for care, was used to assess the occurrence of unmet needs for care (see Additional file2: Appendix for details). The questionnaire was pre-tested to assess the face validity and understanding of the questions with a limited set of patients (n = 10). This project has received full ethical approval from the Ethics review committee from the Montreal Health and Social Services Agency.

Rates of missing responses remained below 1 % for most questions with the exception of household income which showed a slightly higher rate of non response of 8 %. Usual sources of care were identified by nominal identification of the principal healthcare provider during the two years preceding the survey. Organisations for which patients reported an affiliation were classified according to their official denomination type (solo provider, private group practice, family medicine group, local community health centre, emergency department or specialised source of care). People reporting no utilisation of services over a two-year period were classified as not having a usual source of care despite reporting having a personal family doctor.

Variables significant at the 80 % significance level on bivariate analyses or conceptually fundamental to the analyses were included in multiple regression models. Logistic regression models of presence or absence of reporting of unmet healthcare needs (Yes/No) among all respondents were developed by the introduction of blocks of variables. The first block included demographic information (age, sex, education, occupation). The second block comprised economic status variables. The third block included health status variables. The fourth block included affiliation to a family doctor and type of usual source of care. A parsimonious model was obtained by excluding non significant variables that did not affect the remaining variables. All analyses were weighted by attributing the inverse probability of selection of participants in order to account for unequal probabilities of sampling which arise from the stratified two-stage sampling (local area sampling, intra-household selection) and for age and sex distribution compared to census data (post-stratification).

## Results

**Figure 1:**
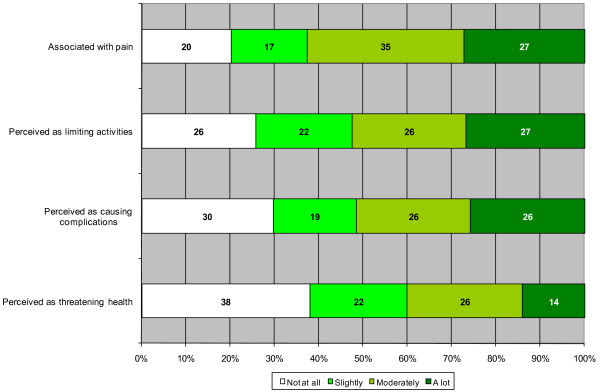
Nature of health problems related to unmet needs for healthcare.

In the six months preceding the survey, 18 % of respondents reported an unmet healthcare need. Figure 1 shows the nature of the health problems for which an unmet healthcare need was reported. From this figure, we can see that a fair proportion of people perceived their health problem as serious with 40 % perceiving the problem to be threatening for health *moderately* or *a lot,* and 38 % perceiving it as not threatening at all. The fact that 62 % felt *moderately* or *a lot* of pain and 53 % were *moderately* or *a lot* limited in their activity also highlights the perceived importance of the problems for which needs were not met. Up to 52 % were *moderately* or *a lot* afraid of complications.

**Figure 2:**
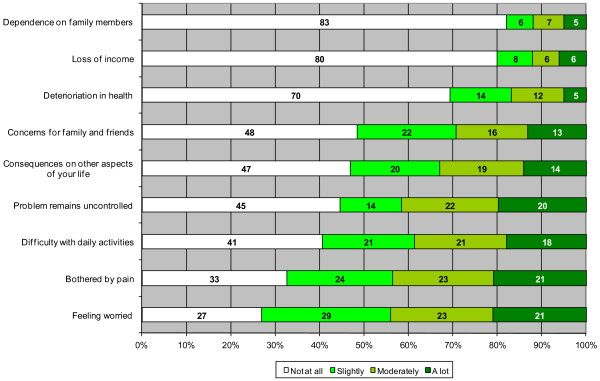
Consequences of unmet needs for healthcare.

In addition, we can see from Figure 2 that a number of adverse consequences were reported as being linked with the unmet healthcare needs in our study. High proportions of people reported moderate or high levels of anxiety (44 %), pain (44 %), unresolved health problems at time of survey (42 %) and difficulties in daily activities (39 %). However, the actual proportion of people reporting deterioration in their health status or loss of autonomy secondary to the health problems remained low. Finally, only 20 % of people with unmet needs for care reported having lost revenues during the illness episode.

**Figure 3:**
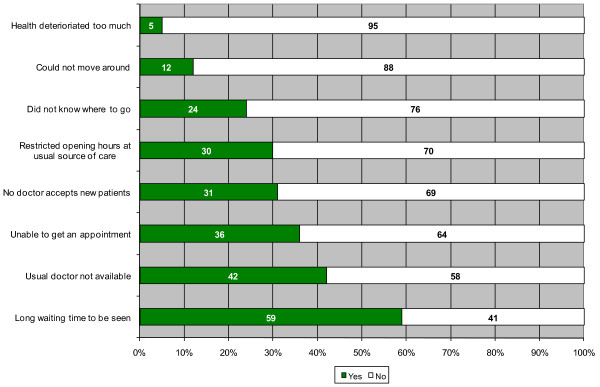
Reported reasons for unmet needs for healthcare.

**Figure 4:**
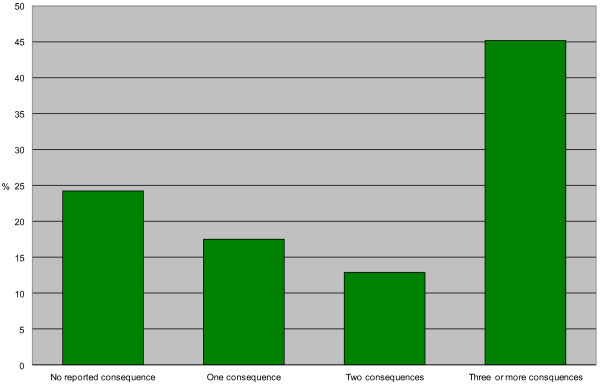
Proportion of people reporting to be strongly or somewhat having experienced none, one, two or three or more consequences of their unmet needs.

Figure 3 shows the reasons perceived by respondents as having contributed to the occurrence of the unmet healthcare need during the study period. Noticeable are factors related to the healthcare system, such as the inappropriate waiting times to be seen, the unavailability of the usual doctor, the impossibility to obtain an appointment, the non availability of doctors accepting new patients and the inappropriate opening hours of the clinic. Individual factors such as not knowing where to go, immobility or health being too deteriorated to enable the person to consult were reported much less often. Figure 4 shows the proportion of people that reported none, one, two, three or more consequences for which they felt to be *affected strongly* or *somewhat*. This reveals an important proportion of people with unmet needs being strongly or somewhat affected on multiple levels.

Table 1 shows the prevalence of reporting unmet needs for care according to various individual and household characteristics and primary care affiliation. From these bivariate associations, we can see that unmet healthcare needs reporting diminishes with increasing age and improvement in reported health status while it increases with education and perceived poverty status. People with a family doctor report lower levels of unmet healthcare needs. In addition, some variability is found between specific types of usual sources of care. In general, people without a usual source of primary healthcare, those reporting a solo practice and a family medicine group report lower levels of unmet needs in bivariate analyses.

**Table 1:** Bivariate associations of sociodemographic characteristics with reporting unmet needs

		**Sample size**	**Unmet needs (%)**	**p-value**
**Age**	18-44	4670	**24**	0.000
	45-64	3157	**16**	
	65+	1379	**5**	
**Sex**	Female	5311	**19**	0.131
	Male	3895	**17**	
**Language**	French	6886	**19**	0.000
	English	1607	**14**	
	Other	713	**19**	
**Education**	Primary or less	1463	**11**	0.000
	Secondary and professional schooling	3134	**16**	
	College	2216	**21**	
	University	2271	**22**	
**Occupation**	Student	762	**25**	0.000
	Employed	5843	**21**	
	Unemployed/social welfare	405	**19**	
	Inactive (retired/volunteer)	2196	**8**	
**Perceived poverty**	Wealthy	2220	**16**	0.000
	Average	5455	**17**	
	Poor	1322	**23**	
	Very poor	209	**30**	
**Perceived health**	Excellent	1943	**14**	0.000
	Very good	3103	**16**	
	Good	2628	**20**	
	Average	1283	**25**	
	Bad	249	**26**	
**Morbidity**	No reported health problem	3727	**16**	0.000
	Isolated risk factor	1109	**12**	
	Moderate morbidity	2568	**22**	
	Severe morbidity	1553	**22**	
	Co-morbidities	249	**21**	
**Having a family doctor**	No	2674	**26**	0.000
	Yes	6532	**15**	
**Usual source of care**	No usual source of care	1272	**16**	0.000
	Solo provider	943	**16**	
	Family medicine group	1170	**17**	
	Private group practice	3864	**20**	
	Local community health centre	653	**21**	
	Emergency department	282	**29**	
	Specialised source of services	633	**18**	

Table 2 presents the results from the multiple regression analysis. The final model confirms the decreasing gradient of unmet healthcare needs with increasing age, controlling for other factors. It suggests that females tend to report more unmet healthcare needs than males. Surprisingly, language spoken at home is associated with varying odds of reporting unmet healthcare needs with French being associated with more unmet needs compared to other languages. As found in previous studies, higher education is associated with more perceived unmet needs, as is regular employment status. Vulnerability variables related to perceived poverty and poorer health status were strong predictors of unmet need for care, a gradient of effect being present in both cases. The poorest and sickest have the highest propensity to declare unmet healthcare needs.

**Table 2:** Factors associated with reporting unmet needs, weighted logistic regression

**Independant variables**		**Odds ratios**	**Confidence intervals*(95 %)***		***p value***
**Age***(Ref.: 65 and +)*	18-44	**4.691**	3.424	6.426	< 0.000
	45-64	**2.821**	2.085	3.817	< 0.000
**Sex***(Ref.: Male)*	Female	**1.156**	1.025	1.303	0.018
**Language***(Ref.: French)*	English	**0.698**	0.592	0.823	< 0.000
	Other	**0.758**	0.619	0.929	0.007
**Education***(Ref.: University)*	Primary or less	**0.541**	0.434	0.673	< 0.000
	Secondary and prof.schooling	**0.608**	0.523	0.707	< 0.000
	College	**0.812**	0.697	0.945	0.007
**Occupation***(Ref.: Employed)*	Student	1.033	0.855	1.249	0.733
	Unemployed/social welfare	**0.604**	0.446	0.818	0.001
	Inactive (retired/volunteer)	**0.612**	0.491	0.763	< 0.000
**Perceived poverty***(Ref.: Wealthy)*	Average	**0.718**	0.591	0.873	0.001
	Poor	**0.778**	0.658	0.921	0.004
	Very poor	1.293	0.883	1.892	0.186
**Perceived health***(Ref.: Excellent)*	Very good	**0.583**	0.487	0.697	< 0.000
	Good	**0.685**	0.591	0.793	< 0.000
	Average	**1.519**	1.273	1.813	< 0.000
	Bad	**1.555**	1.104	2.190	0.012
**Morbidity***(Ref.: No reported health problem)*	Isolated risk factor	0.986	0.793	1.227	0.9000
	Moderate morbidity	**1.915**	1.651	2.222	< 0.000
	Severe morbidity	**1.877**	1.581	2.229	< 0.000
	Co-morbidities	**2.727**	1.880	3.955	< 0.000
**Having a family doctor***(Ref.: No)*	Yes	**0.553**	0.485	0.631	< 0.000
**Usual source of care***(Ref.: No usual source of care n = 1272)*	Solo provider	1.023	0.781	1.340	0.877
	Private group practice	1.279	0.937	1.745	0.121
	Family medicine group	1.151	0.834	1.588	0.393
	Local community health centre/Teaching unit	1.232	0.974	1.558	0.082
	Specialised source of care	**1.486**	1.031	2.144	0.034
	Emergency department	1.333	0.992	1.790	0.056

Legend: Figures in bold are statistically significant. All data weighted to correct for differential probability of inclusion. All odds ratios controlling for other variables in the multiple regression model.

Finally, our results show that affiliation to a given source of care has a strong impact on the occurrence of unmet needs for care, even when other individual factors are taken into account. Having a family doctor is associated with much lower odds of unmet needs for care. In contrast, identifying a usual source of primary care is associated with higher levels of unmet needs for care in certain types of organisations. Only in specialist clinic models are unmet needs for care higher than in the population reporting no usual source of care. Despite showing a higher odds ratio of reporting unmet needs, controlling for other factors, emergency department users did not differ statistically in regression models.

## Discussion

### Increasing importance of unmet needs for care

Our study reports high levels of unmet needs in two regions of Québec province, Canada. Nearly one person out of five reported an unmet need during the six months preceding the survey. This prevalence is higher than previously reported rates in the Canadian context for a 12 month period [1]. This difference could represent higher levels of unmet healthcare needs in the specific context under study. However, our results are in line with previous reported increases in unmet needs in the last 5 years among Canadian provinces as reported in Additional file 2: Appendix. In addition, a shorter period of recall might prove more accurate with regards to identifying instances of unmet needs. We may also have identified as unmet needs ‘problems which will eventually need to be dealt with in the following weeks’ and this might explain our slightly higher level compared to studies with longer recall periods.

Furthermore, our study highlights the importance that these unmet needs have for the public. The problems for which an unmet healthcare need is reported are perceived to have an impact on lives. This suggests that these reasons cannot be dismissed as problems that are *not serious enough for the person to seek care*. Attention should be paid to unmet needs by healthcare policy makers and managers as they could reflect underlying problems in access to healthcare in Canada. Recent international surveys have confirmed the acute problems of access to healthcare in the Canadian context particularly in the province of Quebec [23]. However, increasing discontent with the healthcare system and increasing media coverage about care gaps and safety problems could also encourage the reporting of unmet healthcare needs. The extent to which the increase in unmet healthcare needs results from variations in peoples’ perception, from increasing needs among the population, or from real barriers to access care remains to be assessed and further studies should try to disentangle these specific effects in understanding these phenomena. For practitioners, our study might suggest that unmet needs for care remain an important indicator of the clinics’ capacity to address their patients’ needs through the level of affiliation that they develop with their patients.

### Economic vulnerability and unmet needs in a universal system

Our study is amongst the first, in the Canadian context, to identify perceived poverty as a factor associated with the reporting of unmet healthcare needs. Previous studies had suggested that the universal coverage guaranteed by the Canada health act through a taxation-based funding mechanism effectively prevents such inequity. Given the current context of increasing private costs of healthcare services in Canada, such as the private financing of diagnostic services and increasing drug costs, this result is not surprising. Access to free public healthcare services is associated with payment for drugs given on site, diagnostic procedures, or medical services in up to 28 % of cases; 33 % of people report losing revenue when consulting their primary care clinic [24]. Both utilisation and non-utilisation of healthcare are associated with costs; in our study, 20 % of people with unmet needs had lost revenues (data not shown).

Accumulated knowledge about the direct and indirect costs involved in consulting medical services could prevent poorer households to actually seek otherwise *free* medical services. The fact that absolute household income was rejected from the final analysis, with perceived poverty capturing most of the association, highlights the difficulty in relying on income to assess inequalities and equity. In our study, a fair proportion of people with low household income did not perceive themselves as poor. These include students and retirees for which current income is a poor predictor of economic status. A strength of this study is its use of various measures of economic status, including income and assets but also relative economic status.

Our study also found that unmet needs for care increases with educational status, in line with what was found from previous studies highlighted in the introduction. The inverse association of unmet needs with perceived poverty (the poor reporting more unmet needs) and with education (the less educated reporting less unmet needs) is also of interest. Although it is usually thought that the poor tend to be less educated, our study suggests that population studies in metropolitan areas challenge this assumption. Students and immigrants, who show higher levels of education, tend also to report lower income. The retired, even if well educated, tend to report lower income (despite accumulated assets). These complex relationships between income, education, and unmet needs could explain why perceived poverty was the most important individual economic status characteristic related to unmet needs in our study. In fact, correlations between the different economic status measures were moderate (for example, reported income moderately correlated with perceived economic status (Pearson 0.463) and reported assets (Pearson 0.339) (full data correlation matrix available upon request).

### Unmet healthcare needs as a mirror of PHC organisation and accessibility

Among the strongest associations found in this study is the affiliation with a primary healthcare provider through having a family doctor. Contrasting with healthcare user studies suggesting relatively few accessibility problems in primary healthcare - at least on the basis of the geographical and temporal access measures available - our population-based study could assess the impact of not having a pre-determined entry into the healthcare system on access to healthcare at times of perceived need. This is probably where Canada, and especially the province of Québec, has been lagging behind other highly industrialised nations [25,26]. In addition, various factors such as modifications to the age and sex composition of the medical workforce, changes in the average amount of time spent in clinical activities in primary or secondary care settings, or specific organisational characteristics of the primary healthcare infrastructure, could explain this situation.

In our study, 30 % of respondents declared not having a family doctor and up to 14 % did not identify a usual source of care in the last two years. However, the fact that people without a usual source of care also report lower unmet needs suggests that a fair proportion of people without a usual source also present few perceived care needs. This highlights the fact that, with regards to need for care, the gaps in affiliation do not necessarily translate into accessibility problems for a portion of unaffiliated people. Nevertheless, our study highlights the need, for clinical practice contexts, to foster a strong link between patients and providers in order to reduce unmet needs.

Organizational factors seem to be strong determinants of unmet needs for care as reflected by the reasons reported for not obtaining care. Three respondents out of ten identified *not finding a doctor accepting new patients* as a contributor to their unmet healthcare need. Other organisational factors related to unmet healthcare needs suggest that primary healthcare delivery models have a lot to do in preventing unmet healthcare needs. Affiliation with a primary care clinic seems necessary, but certainly not sufficient to ensure access. Our study did not find a difference with regards to the type of organisation reported as usual source of care, with the exception of solo providers. This might suggest that various types of organisations are able to provide an affiliation with a primary care provider, the single most important factor our study identified as protecting from unmet needs.

Our results also suggest that use of a specialist clinic or a hospital emergency department for usual care does not provide an opportunity to develop an affiliation with a primary healthcare provider and, as a result, does not protect against unmet needs for care, despite its organisational characteristics promoting constant access. Future studies could assess which organizational mechanisms could be set in place to ensure that regular users of emergency departments, or individuals for which it represents the sole source of care, are provided with alternative follow up in primary healthcare settings to prevent future unmet needs for care and consequent utilisation of emergency departments.

Our study could not find differences among specific types of primary healthcare organisations. Solo providers, private group practice, family medicine groups and local community health centres did not statistically differ from each other with regards to the proportion of patients reporting unmet needs. The relatively small number of people affiliated with local community health centres and family medicine groups probably resulted in lack of power to detect differences among these models. However, the results of the *p-*values also suggest that more stringent criteria to assess statistical significance would suggest no statistical difference according to usual source of care. In addition, this study could not fully assess the latter model (Family medicine groups), as it was being conducted at the start of the PHC reform. Further studies, following the more recent increase in population coverage by the emerging family medicine groups could provide stronger evidence of trends indicated in this study.

It is important to highlight here that affiliation with a primary healthcare provider is also strongly associated with age and with the types of primary healthcare organization to which people are associated. People over the age of 65 reported having a family doctor in about 90 % of cases and people reporting a solo provider as their usual source of care also have higher reporting of having a family doctor [24]. This could partly explain the fact that our study could not find differences between types of primary healthcare organizations, controlling for other factors including affiliation with a family doctor. The impact of types of primary healthcare could to a large extent be mediated through the capacity of an organisation to provide a family doctor – an affiliation with a provider that takes responsibility for your care. In fact, Family medicine groups, a model consistent with the patient-centred medical home concept, were associated with lower unmet needs in bivariate analyses. The disappearance of this association can be attributed to a great extent on the impact of having a regular family doctor. Family medicine groups and solo-providers are associated with the highest levels of affiliation with a personal primary care doctor.

### Study strengths and limits

Our study is among the first population-based studies with a large representative sample to have identified and classified usual sources of care. This study provides a large (representative for sex and age composition) sample of the population enabling the reporting of rates of unmet needs and other aspects of the experience of care at the local level, and, more importantly with a link with the usual provider of primary care. Such a large sample was required to provide a good fit between surveyed organisations in the regions and the respondents selected in a random sampling fashion. Smaller sample size would not have enabled us to analyse the impact of organisational models, and related organisational characteristics, at the population level. It should also be mentioned that such a large sample size increase the risk of finding associations which in reality do not convey a clinical significance. However, the associations found in the paper are in line with previously published studies on unmet needs and the association with types of organisations, which is the distinct contribution of this paper, did not reveal significant but weak odds ratios, which would have been suggestive of a statistically significant but minor association.

The two selected regions are representative of Quebec province for age and sex distribution, are the two most populous (accounting for 43 % of the province’s population and approximately half of all primary healthcare organisations in the province) and sprawl across a diverse array of contexts, including metropolitan, suburban, small town and rural areas. The random selection of households and respondents, and the use of sampling and post-stratification weights, ensures a representative sample of the population and the provision of robust estimates whilst taking into account the sampling design. In addition, the questionnaire developed for the study is probably among the most extensive questionnaires on unmet healthcare needs currently in use.

However, our design remains a cross-sectional study. This type of study design is always subject to recall bias and difficulty in assessing the directionality of the associations found. In addition, the assessment of unmet healthcare needs remains a subjective personal evaluation, which will vary among individuals. Personal expectations towards the healthcare systems and towards health can also be associated with the perception of unmet needs, independent of people’s health status and access to healthcare services. The extent to which the reported association is the reflection of these perception biases cannot be assessed or controlled entirely, despite our use of sophisticated methods. Furthermore, other reasons for unmet healthcare that were not measured in our study may also be important and as mentioned earlier, our study occurred at the start of the reform. A longitudinal study looking at the evolution of unmet needs through several years of reform would bring a more definitive understanding of the potential impact of reform models on unmet needs.

## Conclusion

This study reveals increasing trends in reporting unmet needs for care in a Canadian province. In addition, we have identified organisational factors associated with the occurrence of unmet needs for care. Affiliation with a primary healthcare provider is a prime factor associated with lower levels of unmet needs. Decision-makers and managers should be attentive to the emerging trend in unmet needs for care, especially for vulnerable populations. New types of primary healthcare delivery could promise better addressing the needs of populations; however, our study suggests few differences in the occurrence of unmet needs for care across different types of PHC organisations or sources of care. The factors associated with reduced unmet needs could be applicable to every type of clinical settings in the primary care sector. Further evaluations should address this issue to guide future reform models and innovations in primary healthcare.

## Endnotes

^a^Among the 25278 phone numbers generated by the Random Digit Dialling method, 14749 turned-out to be valid numbers. Among these valid number, 2189 households did not accept to participate in the survey and 1164 selected individuals from participating households refused to participate, 366 were not able to answer (language problems or incapacity) and 649 could never be contacted by phone. Up to 140 calls were made to try to reach valid numbers.

^b^ The response rate has been calculated according to the method used by the Association de l'industrie de la recherche marketing et sociale (AIRMS Québec). This method parallels the Data Collection Response Rate Calculation recommended by Statistics Canada in its Standards and Guidelines for Reporting of Non-Response.

## Competing interests

The authors declare that they have no competing interests.

## Authors’ contributions

JFL, RP, MH and DR participated in the design of the study, contributed to the literature review, were involved in the interpretation of the data and critically revised the manuscript for important intellectual content. All these authors read and approved the final manuscript. CK, BS and AP were responsible for the data processing and the statistical analyses.

## Funding sources

Canadian Health Services Research Foundation and Canadian (CHSRF), Institutes of Health Research (CIHR), Fonds de la recherche en santé du Québec.

## Previous presentations

Levesque J-F., Pineault R, Kapetanakis C, Hamel M, Roberge D, Robert L, Simard B. (2009). Primary Care Affiliation And Unmet Needs for Healthcare Services for Vulnerable Populations: Insights From a Population-based Survey In Quebec Province. North American Primary Care Research group Annual Meeting, Montreal, Quebec, Canada. 14–18 November 2009.

Levesque, J-F., Pineault, R., Kapetanakis, C. (2007). Primary Care Affiliation and Unmet Needs for Care for Vulnerable Populations: Results of a Population-based Survey in Quebec Province. Conférence de l'Association canadienne pour la recherche sur les services et les politiques de la santé (ACRSPS), Toronto, Ontario, Canada. 12–14 June 2007.

## Pre-publication history

The pre-publication history for this paper can be accessed here:

http://www.biomedcentral.com/1471-2296/13/66/prepub

## Supplementary Material

Additional file 1 **Appendix 1.** Unmet needs in recent international studies [27,28]. Click here for file

Additional file 2 **Appendix 2.** Unmet needs for care section items. Click here for file
