# Supplementary material for: Emerging organisational models of primary healthcare and unmet needs for care: insights from a population-based survey in Quebec province
Source: BMC Fam Pract. 2012 Jul 2;13:66. doi: 10.1186/1471-2296-13-66 (PMC3431245; doi:10.1186/1471-2296-13-66)
Supplement: Additional file 1 — Appendix 1. Unmet needs in recent international studies [27,28]. [file 1471-2296-13-66-S1.pdf]

Appendix 1. Unmet needs in recent international studies.

**Source**	**Reference period**	**Target population and year of survey**	***Proportion***
Sibley LM and Weiner JP (2011). An evaluation of access to healthcare services along the rural-urban continuum.	Past 12 months	20 years and over, Canada, 2003	Ranging between 10.2 and 12.7% across rural and urban settings
McGrail KM, Van Doorslaer E et al. (2009). Income-related health inequalities in Canada and the United States: A decomposition analysis.	Past 12 months	18 years and over, Canada and United States, 2002-2003	Canada: 8.4%<br>United States: 10.6%
Lasser KE, Himmelstein DU and Woolhandler S (2006). Access to care, health status, and health disparities in the United States and Canada: Results of a cross-national population-based survey.	Past 12 months	18 years and over, Canada and United States, 2002	Canada: 10.7%<br>United States: 13.2%
Kasman NM and Badley EM (2004). Beyond access: who reports that health care is not being received when needed in a publicly-funded health care system?	Past 12 months	15 years and above, Canada, 2000-2001	12.8%
Pagan J and Pauly M (2006). Community-level uninsurance and the unmet medical needs of insured and uninsured adults	Past 12 months	18-64 years, United States, 2000-2001	Insured: 7%<br>Uninsured: 18%
Shi L and Stevens GD (2005). Vulnerability and unmet health care needs: The influence of multiple risk factors	Past 12 months	18 years and over, United States, 2000	Between 2% and 20%, depending on the number of risk factors
Wu Z, Penning MJ and Schimmele CM (2005). Immigrant status and unmet health care needs	Past 12 months	18 years and over, Canada, 2000-2001	11.6% for immigrants and 13.6% for non-immigrants
Diamant AL, Hays RD et al. (2004). Delays and unmet need for health care among adult primary care patients in a restructured urban public health system	Past 12 months	Patients 18 years and over who had used primary care services, United States, 1999	25%
Sanmartin C, Houle C et al. (2002). Besoins non satisfaits de soins de santé : évolution	Past 12 months	12 years and over, Canada, 1994-2001	4.2% in 1994-95,<br>5.1% in 1996-97,<br>6.3% in 1998-99,<br>12.5% in 2000-01
Donelan K, Blendon RJ et al. (1999). The cost of health system change: Public discontent in 5 nations	Past 12 months	18 years and over, 5 countries, 1998	Canada: 10%<br>Australia: 8%<br>Great Britain: 10%<br>New-Zealand: 12%<br>United States: 14%
