# Supplementary material for: Emerging organisational models of primary healthcare and unmet needs for care: insights from a population-based survey in Quebec province
Source: BMC Fam Pract. 2012 Jul 2;13:66. doi: 10.1186/1471-2296-13-66 (PMC3431245; doi:10.1186/1471-2296-13-66)
Supplement: Additional file 2 — Appendix 2. Unmet needs for care section items. [file 1471-2296-13-66-S2.pdf]

Appendix 2. Unmet needs for care section items.

Item	Introduction	Question	Response categories
Q_D1	The next questions are about the health services you needed and did not get.	During the last 6 months, did you feel you needed to see a doctor for a health problem but didn't see one?	1=Yes<br>2=No<br>95=Does not know<br>97=Refused
Q_D2	For what reason did you want to see a doctor?	Was it...	1=For an urgent health problem<br>2=For an non urgent health problem<br>3=To pass routine exams<br>5=To receive results from testsor exams, or from a consultation to a specialist<br>7=To have a form filled out<br>90=Other reason<br>97=Refused
Q_D2.1	Was this a known problem or was it a new problem ?		1=Known problem<br>2=New problem<br>97=Refused
Q_D3	Do you still have this problem?		1=Yes<br>2=No<br>95=Does not know<br>96=DNA<br>97=Refused
Q_D4	Tell me if the following questions apply a lot, moderately, slightly or not at all.?	Was this problem causing you pain?	1=A lot<br>2=Moderately<br>3=Slighlty<br>4=Not at all<br>95=Does not know<br>96=DNA<br>97=Refused
Q_D5		Was this problem threatening your health?	
Q_D6		Were you afraid of having complications if this problem was not treated quickly ?	
Q_D7		Was the problem for which you wanted to consult limiting your activities?	
Q_D8	What were the reasons you did not see a doctor? Was it because…	You received an appointment but have not seen the doctor yet.	1=Yes<br>2=No<br>95=Does not know<br>96=DNA<br>97=Refused
Q_D9		You didn't know where to go to see a doctor.	
Q_D10		You couldn't get an appointment.	
Q_D11		You couldn't move around to actually go see a doctor.	
Q_D12		You couldn't find a doctor who is taking new patients.	
Q_D13		Your usual doctor wasn't available at the time you needed him or her.	
Q_D14		The waiting time before seeing a doctor was too long.	
Q_D15		The office hours during which you could see a doctor did not suit you.	
Q_D19		Your health status deteriorated too much so that you could not go see your doctor.	
Q_D20	Still thinking about the problem for which you wanted to see a doctor, tell me if you agree strongly, somewhat, a little or not at all with each of the following statements:<br><br>Since the problem appeared…	Your health has worsened.	1=Strongly<br>2=Somewhat<br>3=A little<br>4=Not at all<br>95=Does not know/<br>97=Refused
Q_D21		This situation caused you to feel worried, stressed or anxious.	
Q_D22		This situation caused some of your family members and friends to feel worried, stressed or anxious.	
Q_D23		You were bothered by the pain.	
Q_D24		You had difficulty doing your daily activities.	
Q_D25		This situation had negative consequences on other aspects of your life.	
Q_D26		You lost some income.	
Q_D27		This situation made you dependent on family members or friends.	
Q_D28		You still have not succeeded in managing your health problem.	
